# Considerations Regarding Public Use of Longevity Interventions

**DOI:** 10.3389/fragi.2022.903049

**Published:** 2022-04-25

**Authors:** Yasmine J. Liu, Rebecca L. McIntyre, Georges E. Janssens

**Affiliations:** Laboratory Genetic Metabolic Diseases, Amsterdam Gastroenterology, Endocrinology, and Metabolism, Amsterdam Cardiovascular Sciences, Amsterdam UMC location University of Amsterdam, Amsterdam, Netherlands

**Keywords:** aging, public, interventions, geroprotectors, biological age, chronological age

## Abstract

Public attention and interest for longevity interventions are growing. These can include dietary interventions such as intermittent fasting, physical interventions such as various exercise regimens, or through supplementation of nutraceuticals or administration of pharmaceutics. However, it is unlikely that most interventions identified in model organisms will translate to humans, or that every intervention will benefit each person equally. In the worst case, even detrimental health effects may occur. Therefore, identifying longevity interventions using human data and tracking the aging process in people is of paramount importance as we look towards longevity interventions for the public. In this work, we illustrate how to identify candidate longevity interventions using population data in humans, an approach we have recently employed. We consider metformin as a case-study for potential confounders that influence effectiveness of a longevity intervention, such as lifestyle, sex, genetics, age of administration and the microbiome. Indeed, metformin, like most other longevity interventions, may end up only benefitting a subgroup of individuals. Fortunately, technologies have emerged for tracking the rate of ‘biological’ aging in individuals, which greatly aids in assessing effectiveness. Recently, we have demonstrated that even wearable devices, accessible to everyone, can be used for this purpose. We therefore propose how to use such approaches to test interventions in the general population. In summary, we advocate that 1) not all interventions will be beneficial for each individual and therefore 2) it is imperative that individuals track their own aging rates to assess healthy aging interventions.

## Introduction

Increased life expectancy in human populations has been accompanied by increased rates of age-related diseases. This can include non-communicable age-related diseases, including cardiovascular diseases, neurodegenerative diseases, chronic kidney disease, and metabolic diseases such as obesity and type 2 diabetes. Advanced age also results in an increased susceptibility to communicable diseases, resulting in potentially severe clinical outcome upon influenza or COVID-19 infections. Aging has long been considered to be a passive process that could not be reverted or slowed down. However, it is now clear that the aging program is actively regulated (López-Otín et al., 2013), a discovery that has been accompanied by a paradigm shift in thinking; that regulating aging can reduce the burden of age-related diseases.

Although it has been a long time coming, the World Health Organization now defines aging as a disease in the latest version of the International Classification of Diseases (ICD-11, code ‘Ageing-related’ XT9T) (Khaltourina et al., 2020). The increased recognition of aging as a disease is meaningful for the development of future therapeutic interventions or strategies targeting aging and aging-related diseases (The Lancet Diabetes and End, 2018). Several classes of compounds that extend lifespan in model organisms hold great promise for clinical application in targeting human aging. In a recently published review, compounds including mTOR inhibitors, senolytics, metformin, acarbose, spermidine, NAD^+^ boosters, and lithium were deemed worthwhile to test in humans for their effects on healthspan (Partridge et al., 2020). Those compounds were selected given their reproducible pro-longevity effects in animal models, conserved mechanisms of action, significant amelioration of human biomarkers of aging, and potentially good safety profiles (Moskalev et al., 2016; Partridge et al., 2020). These factors therefore make up a primary set of criteria for evaluation of compounds for translatability to humans (Moskalev et al., 2016; Partridge et al., 2020).

Many longevity interventions have been described in model organisms, for example, the DrugAge database of aging interventions lists over 400 such compounds (Barardo et al., 2017; Janssens and Houtkooper, 2020). Yet, a major difficulty remains in gathering evidence that these compounds can influence the rate of biological aging in humans, other than by conducting an actual clinical trial. In this work, we advocate a parallel, non-mutually-exclusive route for selecting longevity interventions to test, starting from human population data and assessing rates of biological aging therein. We go on to illustrate how a variety of factors will influence a compound’s effectiveness as a longevity intervention, focusing on metformin as a case study. Finally, we describe how tracking the biological aging process can be used to test longevity interventions in the population, and is in fact a requisite for personalized longevity interventions.

### Population Sourced Identification of Longevity Interventions

Recently, our team demonstrated that calculating the biological aging rate in a population can serve to identify factors associated with age deceleration (McIntyre et al., 2021). In this recent study, we used wearable device movement patterns to define a biological aging score, and checked for food components and drugs that were associated with age deceleration (McIntyre et al., 2021). We identified fiber, magnesium, and vitamin E as food components and the use of alpha blocker doxazosin as a pharmaceutical associated to younger biological age (McIntyre et al., 2021). Remarkably, we demonstrated that treating *C. elegans* with doxazosin could increase their aging health (measured by mobility and motility) in addition to their lifespan (McIntyre et al., 2021).

**FIGURE 1:**
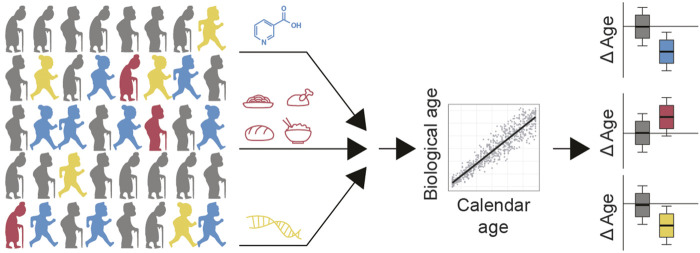
Strategy for identifying candidate longevity interventions in humans. Population level data would ideally contain information on treatments that people take, e.g. supplements and drugs, as well as their nutrition patterns and genetics. Calculating biological age of each participant and comparing this to their calendar age would identify which of these factors potentially slow aging (e.g. blue and yellow) compared to interventions which may accelerate aging (e.g. red). Factors that slow aging (blue, yellow) or accelerate aging (red), may comprise nutrients, drugs, exercise routines, other lifestyle factors and/or combinations thereof. These would become candidate therapies to test in a prospective cohort.

The approach we describe, consisting of a retrospective analysis on the rate of aging, and identifying factors associated with age deceleration, is a general approach that can be employed throughout many large human cohort datasets. While our study used wearable device accelerometer data to determine a biological aging score, biological age can also be assessed using a wide variety of other aging scores, including DNA methylation-based, transcriptomics-based, proteomics-based, and metabolomics-based molecular aging clocks (Jylhävä et al., 2017; Zhavoronkov and Mamoshina, 2019; Galkin et al., 2020; Robinson and Lau, 2020), in addition to other phenotypic parameters, e.g. blood biochemistry markers (Levine et al., 2018). Fortunately, these types of datasets are increasingly becoming available in large and thoroughly characterized human population studies. Following this, calculating biological age for each individual, and comparing this to their calendar age, can identify individuals with decelerated aging rates. Finding what factors, whether nutritional supplements, pharmaceuticals, food components, exercise routines, or genetics, that associate with younger age, can provide candidate interventions for follow up in prospective studies (Figure 1).

Studies, in addition to our own, have begun to apply such approaches and in doing so have identified candidate interventions with the potential to promote healthy aging in humans. For example, using an epigenetic aging clock, higher quality diets were related to decelerated aging (Kim et al., 2022). Furthermore, looking at telomere lengths and mortality data, it was found that Mediterranean diets may also decelerate aging (Crous-Bou et al., 2014). Using an epigenetic aging clock, it was also seen that certain mood stabilizers (including lithium carbonate, sodium valproate, and carbamazepine) are associated with decelerated aging (Okazaki et al., 2020). In line with this, studies in model organisms have demonstrated that this class of drugs can extend lifespan (Evason et al., 2008; Castillo-Quan et al., 2016; Nespital et al., 2021). Additionally, analysis of survival data alone can provide insights into factors positively affecting healthy longevity. For example, the antidiabetic drug metformin, discussed as a case-study below, was identified in such an analysis. Metformin users had lower mortality rates than non-diabetic age-matched controls, suggesting metformin was interveneing in the aging process (Bannister et al., 2014). Taken together, identification of decelerated aging factors through reanalysis of human datasets, whether drugs, diets, or otherwise, may offer a high likelihood of translatability to humans.

Population level data would ideally contain information on treatments that people take, e.g. supplements and drugs, as well as their nutrition patterns and genetics. Calculating biological age of each participant and comparing this to their calendar age would identify which of these factors potentially slow aging (e.g., blue and yellow) compared to interventions which may accelerate aging (e.g. red). Factors that slow aging (blue, yellow), or accelerate aging (red), may comprise nutrients, drugs, exercise routines, other lifestyle factors and/or combinations thereof. Those found to slow aging would become candidate therapies to test in a prospective cohort.

### Considerations for Use of Longevity Compounds, a Case Study of Metformin

Metformin is a biguanide drug widely prescribed for the treatment of type 2 diabetes (T2D) worldwide (Adak et al., 2018). Metformin has been used in the clinic for over 6 decades and has an excellent safety record thus far. The medical history of metformin dates back to the 17th century, where the extracts of French lilac (*Galega officinalis*), which contains a metformin-like guanidine compound, was used as an herbal remedy to treat intense urination (Witters, 2001). This was the typical symptom of the disease that later came to be known as diabetes mellitus (Witters, 2001). In 1994, metformin was approved in the U.S. by the FDA to treat type 2 diabetes and is now the most prescribed anti-diabetic medication worldwide, taken by over 150 million people each year (He and Wondisford, 2015). Metformin is an effective glucose-lowering agent while also increasing peripheral sensitivity to insulin, thereby reducing the risk of all diabetes-related complications including death (Rena et al., 2017). Despite the long history and widespread use in the clinic, the modes of metformin’s action underlying these favorable effects are complex and remain elusive. Metformin is not metabolized in humans or animals and is eliminated unchanged by renal clearance (He and Wondisford, 2015). After oral administration, approximately 70% of metformin is absorbed from the small intestine and then delivered directly to the liver where it acts primarily to reduce hepatic gluconeogenesis (He and Wondisford, 2015). In addition to the liver, the intestines are also identified as an important target organ for metformin’s actions. Metformin can stimulate anaerobic glucose metabolism in enterocytes, suppress hepatic glucose production via liver-gut-brain crosstalk (Duca et al., 2015), and alter the gut microbiome in ways that contribute to antihyperglycemic effects, weight loss and inflammation suppression in individuals with T2D (Forslund et al., 2015; Wu et al., 2017).

Metformin is not only used in T2D management but also has gained significant attention as a potential geroprotective agent. Administration of metformin to various model organisms, including *C. elegans* (Onken and Driscoll, 2010) and mice (Martin-Montalvo et al., 2013), can mitigate aging, improve health and thereby increase lifespan through mechanisms beyond blood glucose regulation. The favorable effects of metformin on health and lifespan have been attributed to AMPK activation (Onken and Driscoll, 2010), mitohormesis (De Haes et al., 2014), the lysosomal pathway (Chen et al., 2017), and alterations of the microbiome, specifically by changing microbial folate and methionine metabolism (Cabreiro et al., 2013). In mice, metformin has been shown to extend lifespan and improve health in both short- and long-lived mouse models (Anisimov et al., 2005; Anisimov et al., 2008; Martin-Montalvo et al., 2013; Anisimov et al., 2015), yet the effects seen in long-lived mouse strains appear to be more modest. Some of these studies also suggest a greater health benefit of metformin in female mice (Anisimov et al., 2005; Anisimov et al., 2008; Anisimov et al., 2015). In spite of ample evidence that supports the protective effect of metformin against aging, a study conducted by the Aging Interventions Testing Program reported a non-significant effect of metformin on the lifespan of outbred mice (Strong et al., 2016). The authors suggested that the lack of benefit may be attributed to the doses used in the study (Strong et al., 2016). Given that the outbred mice have been deemed to be genetically heterogenous, these results also indicate that the lifespan response to metformin is subject to genetic variations. Therefore, individualized and precision approaches are needed to implement metformin in aging.

Nonetheless, a consensus exists that metformin targets and delays aging (Barzilai et al., 2016). Its administration in humans provides overall protection against age-related diseases, including but certainly not limited to diabetes, as well as, frailty in general. The geroprotective property of metformin can be evidenced by a wealth of retrospective observational studies, in which metformin use in diabetic patients is associated with reduced incidence of cancer (Currie et al., 2009; Libby et al., 2009; Landman et al., 2010; Lee et al., 2011; Monami et al., 2011; Ruiter et al., 2012; Tseng, 2012; Gandini et al., 2014), cardiovascular disease (Kooy et al., 2009; Wang et al., 2017), cognitive impairment (Cheng et al., 2014; Guo et al., 2014; Ng et al., 2014), and all-cause mortality (Turner, 1998; Bannister et al., 2014). Even when compared to age- and sex-matched non-diabetic groups, metformin treated diabetic patients had a slightly longer survival, despite the fact that diabetic patients were obese and had higher levels of morbidity at baseline (Bannister et al., 2014). A meta-analysis study has replicated these findings, reaching a similar conclusion on the protective effects of metformin against all-cause mortality and diseases of aging when compared to the general and non-diabetic population (Campbell et al., 2017). To explicitly answer the question concerning the anti-aging effects of metformin in non-diabetic individuals, the double-blinded, placebo-controlled multicenter trial Targeting Aging with Metformin (TAME) has been devised (Barzilai et al., 2016). TAME is the first so called ‘geroscience’ guided aging outcomes trial designed for metformin and plans to enroll ∼3,000 non-diabetic men and women aged 65–79 years for a 6-years trial period (Barzilai et al., 2016). In contrast to traditional FDA approved trials that only look for a single disease endpoint, TAME has a composite primary endpoint, consisting of stroke, heart failure, dementia, myocardial infarction, cancer, and death (Justice et al., 2018). This is in particular important to evaluate the efficacy of healthy aging drugs, considering the premise that slowing down aging will automatically postpone or eliminate multiple diseases of old age.

Metformin holds great potential to target aging and increase human healthspan. However, when it comes to the widespread use as an anti-aging therapy, some uncertainty still exists regarding whether metformin is effective and safe across the entire population for aging. One concern regards metformin responders versus non-responders, an issue that has been apparent among patients who take metformin for glycemic control (Florez, 2017). Genetic variation in the organic cation transporters that mediate the transport of metformin in body tissues are suggested to account for the differential responses to metformin (Yoon et al., 2013). In spite of this, understanding of the underlying mechanisms remains superficial. Another uncertainty concerns the interaction between metformin and lifestyle modification such as exercise. Although exercise and metformin independently improve metabolic health, and decrease the risk of age-related diseases, the combination may elicit unfavorable antagonistic effects on physiological function (Konopka et al., 2019). An additional consideration regards age of treatment. While metformin can be used safely and is the preferred initial therapy in many older adults with type 2 diabetes (Sue Kirkman et al., 2012), a study in *C. elegans* demonstrated that late-life treatment with metformin exacerbates aging-associated mitochondrial dysfunction and shortens lifespan (Espada et al., 2020). Furthermore, although metformin has been used with an excellent safety record for over 60 years, its long-term use is still associated with some potential side effects. Among them, gastrointestinal disturbances, such as diarrhea, nausea, vomiting, flatulence, and loss of appetite (Adak et al., 2018), occur most frequently. Very rarely, lactic acidosis may also occur upon metformin use, but the risk appears to be highly related to renal function (Connelly et al., 2017). Altogether, precision medicine approaches will be critical to implement metformin intervention as an anti-aging therapy in humans.

### Testing Interventions in the Population

The case of metformin serves to illustrate that the efficacy of even the most promising longevity compounds will be susceptible to a variety of factors particular to the individual (Table 1). Furthermore, it remains unknown whether factors associated with decelerated aging will actually cause decelerated aging in humans. While conducting clinical trials such as the TAME study can directly address this question, this is an expensive (the TAME trial is estimated to cost 75 million USD (De Grey, 2019)) and time consuming option. While this may be the only way forward for drug interventions, other interventions, including supplements, diets, and exercises regimens, may allow for alternative forms of testing.

**TABLE 1:** Overview of selected factors influencing efficacy of metformin.

Factor	Evidence for influence on metformin efficacy
Sex	Certain Studies in Mice Suggest a Greater Health Benefit of Metformin in Females (Anisimov et al., 2005; Anisimov et al., 2008; Anisimov et al., 2015)
Genetics	A non-significant effect of metformin was seen on the lifespan of genetically heterogeneous, outbred mice (Strong et al., 2016). In humans, genetic variations on the organic cation transporters that mediate the transport of metformin in body tissues are accountable for the differential responses to metformin (Yoon et al., 2013; Florez, 2017)
Lifestyle	Exercise and metformin independently improve metabolic health though the combination may elicit unfavorable antagonistic effects on physiological function in older adults (Konopka et al., 2019)
Age	While metformin can be used safely and is the preferred initial therapy in many older adults with type 2 diabetes (Sue Kirkman et al., 2012), a study in *C. elegans* worms demonstrated that late-life treatment with metformin exacerbates ageing-associated mitochondrial dysfunction and shortens lifespan (Espada et al., 2020)
Dose	Variation in metformin dose produces varying effects (He and Wondisford, 2015) and furthermore, the lack of significant lifespan extension of metformin in outbred mice was speculated to be in part attributed to dose (Strong et al., 2016)
Microbiome	Metformin influences the gut microbiome contributing to antihyperglycemic effects, weight loss and inflammation suppression in T2D individuals (Forslund et al., 2015; Wu et al., 2017). Furthermore, in *C. elegens,* metformin changes the microbia folate and methionine metabolism (Cabreiro et al., 2013). Since metformin acts in part through the microbiome, variation between individuals may play a role

**FIGURE 2:**
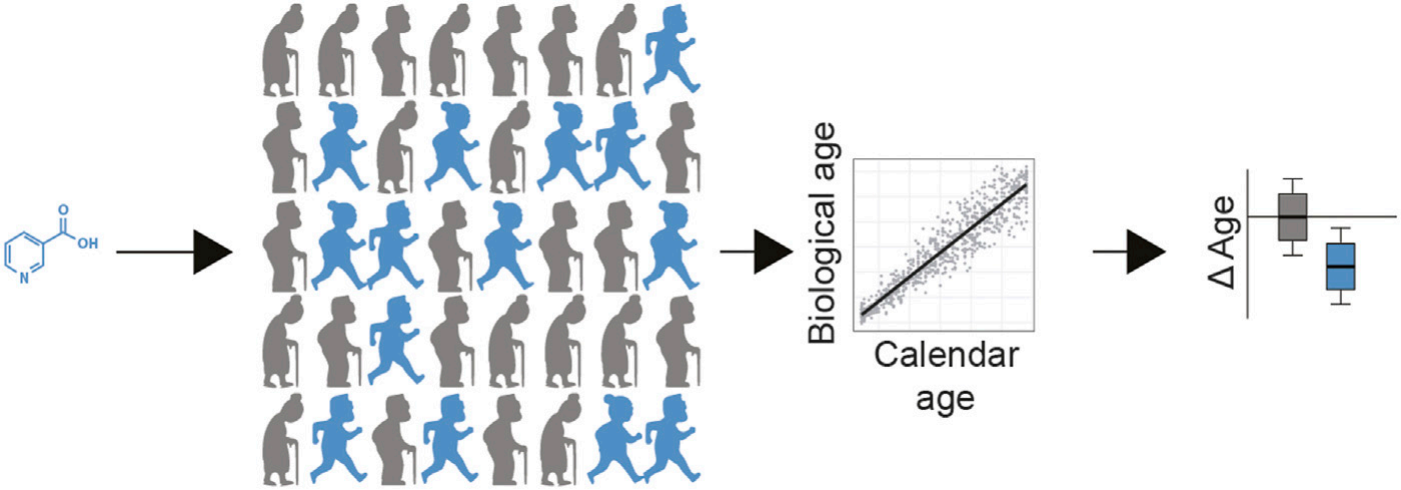
Population level testing of longevity interventions. In this example, a safe nutraceutical can be given to participants (blue = receiving treatment, grey = receiving placebo). After a predefined period of administration, individuals assess their biological age, compared to their chronological age, calculating deltaAge. This can be used to determine if the drug has a statistical effect at decelerating aging at the population level. In addition, personal data of the participants (e.g., sex, genetics, lifestyle, age, genetics, microbiome) can be used to help decipher which sub populations benefit the most from the treatment (not shown).

One possibility is to coordinate open-source public campaigns on such interventions in a systematic manner, to assess in the population whether a particular intervention is working (Figure 2). In theory, individuals may choose to take a safe nutraceutical, exercise regimen, or nutritional diet, which is suspected to slow the aging process. After a pre-defined period of administration, individuals assess their biological age, compared to their chronological age, to determine if the drug has a statistical effect at decelerating aging in the population tested (Figure 2). The most accessible biological aging score may be one based on accelerometers, as we have recently developed (McIntyre et al., 2021), since most individuals possess one in their mobile phones, smart watches, or fitness trackers.

The advantage of the ‘public testing’ scenario described here, is that not only would individuals contribute to testing whether a particular intervention is having statistical effects at a population level, but they would also determine whether they themselves are ‘responders’ or ‘nonresponders’ to the intervention in question. Careful noting of relevant parameters at the onset of the study, including lifestyle factors, sex, age, intervention dose/frequency, and linking with commercially available ‘omics’ technologies such as genomics and microbiome measures, would eventually allow for the disentangling of factors involving efficacy. Ultimately, this would contribute to developing personalized approaches to treating aging for the future.

## Conclusion

Here we 1) describe how biological age prediction can identify factors associated with decelerated aging in large cohort studies, 2) highlight that not all longevity interventions will equally benefit each user, using metformin as a case study, and 3) propose that large-scale controlled public testing of longevity interventions is a theoretical possibility, potentially even a necessity, towards implementing longevity interventions for the public. It is important to note that metformin is not alone in its potential drawbacks as a longevity intervention. For example, dietary restriction, one of the most widely accepted longevity interventions, has either beneficial or detrimental effects on mouse lifespan depending on genetic background (Liao et al., 2010). Furthermore, antioxidants, another popular intervention often praised for healthy aging benefits, can also prevent the benefits of physical exercise in humans (Ristow et al., 2009). Of important consideration too are sex differences. For example, it has been noted that lifespan extension in mice due to (genetically) reduced levels of insulin-like growth factor 1 (IGF-1) or mechanistic target of rapamycin (mTOR) signaling typically favors females, while pharmacological treatments (rather than genetic modifications) generally favors males (Austad and Bartke, 2015). Clearly, as longevity interventions reach the public, it will be of paramount importance to closely track the aging process. Moving forward, we advocate that 1) not all interventions will be beneficial for each individual and 2) it is imperative that individuals track their own aging rates to assess healthy aging interventions.

## Data Availability

The original contributions presented in the study are included in the article/Supplementary Material, further inquiries can be directed to the corresponding author.
